# Ethical and regulatory issues of stem cell-derived 3-dimensional organoid and tissue therapy for personalised regenerative medicine

**DOI:** 10.1186/s12916-022-02710-9

**Published:** 2022-12-27

**Authors:** Alexander R. Harris, Mary Jean Walker, Frederic Gilbert

**Affiliations:** 1 Department of Biomedical Engineering, University of Melbourne, Melbourne, 3010 Australia; 2 Department of Politics, Media, and Philosophy, La Trobe University, Bundoora, Victoria 3086 Australia; 3 Ethics Lab, School of Humanities, University of Tasmania, Hobart, Tasmania Australia

**Keywords:** Stem cell, Regenerative medicine, Ethics, Clinical trials, Preclinical testing, Organoid

## Abstract

**Background:**

Regenerative medicine has the potential to treat genetic disorders and replace damaged or missing tissue. The use of donor or animal tissue raises many well-known issues, including limited tissue availability, the possibility of rejection and patient infection. Stem cell therapy raised hope of overcoming these issues, but created new risks including tumour formation and limited benefit if the desired target tissue does not form. The recent development of 3-dimensional tissues, including organoids, allows the creation of more complex tissues for personalised regenerative medicine.

**Methods:**

This article details the potential health risks of 3-dimensional organoid and tissue therapy versus dissociated stem cell therapy. The current ethical and regulatory issues surrounding 3-dimensional organoid and tissue therapy are presented with a focus on the highly influential FDA and International Society of Stem Cell Research (ISSCR) guidelines.

**Conclusions:**

The potential use of 3-dimensional organoid and tissue therapy may deliver greater patient benefits than other regenerative medicine approaches, but raises new health and ethical risks. Preclinical testing of these therapies will not mitigate some of their risks; they may only be understood after first-in-human trials. The potential irreversibility and high risk of these therapies affects how clinical trials should be structured, including post-trial care for participants.

## Background

Regenerative medicine aims to repair, regrow or replace damaged tissue to restore normal body function. It holds great promise in not only treating patient symptoms, but reversing disease or trauma. Stem cell therapy is one approach to regenerative medicine. Stem cells have the ability to differentiate into specialised cells and have been transplanted into patients in an effort to treat various diseases. Stem cells can be obtained from a donor or be autologous, eliminating any issues from the use of animals in tissue harvesting. Clinical trials have been run for the implantation of stem cells and their derived products. Preclinical trials implanting human- or animal-derived organoids and other 3-dimensional tissue constructs into animals have been performed [1]. It is therefore expected that clinical trials implanting more complex 3-dimensional stem cell-derived tissue constructs (SCTCs) in humans will occur in the near future, including a recent report of autologous intestinal epithelial organoids being implanted into a patient with ulcerative colitis (jRCTb032190207) [2].

This article investigates the ethical and clinical challenges that will impact on the implantation of SCTCs. Should such treatments come to be offered to patients, there may also be ethical questions relating to their regulation and marketing. It is critical that these issues are addressed before clinical trials are begun, to ensure patients are not placed at needless harm, are treated fairly, and that the trials are best structured for maximising clinical and therapeutic benefit.

As the regulations and guidance around this technology are limited, reducing the benefit in comparing regulations across jurisdictions, we will focus on the FDA and International Society of Stem Cell Research (ISSCR) communications, as they have been widely influential in this area. The National Academies of Sciences, Engineering and Medicine have also released guidance for “Human Embryonic Stem Cell Research” (2005 and updated in 2010) which provides no guidance on induced pluripotent stem cells (iPSCs) [3], and “The emerging field of human neural organoids, transplants, and chimeras” (2021) which didn’t include other stem cell-derived tissues and deliberately excluded transplantation of organoids in humans [4]. In July 2022, the European Union released a Proposal [5] to significantly change its regulation of cells and tissues and repeal Directive 2004/23/EC, the cornerstone of such regulation for the last two decades. While these are minimal standards for EU member states, one of the main concerns was that current legislation does not fully protect patients from avoidable risks, and it was unclear if new therapies were being regulated. Working in conjunction with Regulation (EC) 1394/2007, which regulates marketing of Advanced Therapy Medicinal Products [6], the proposal is likely to alter how SCTCs could be offered to patients prior to marketing approval if adopted, but is broadly formulated rather than offering specific advice on SCTCs.

## Potential usage and benefit of 3-dimensional SCTC therapy

SCTCs can be created from embryonic stem cells, adult stem cells or iPSCs [7]. Adult stem cells are limited in the types of tissue they can differentiate into, while iPSCs are often thought to bypass ethical concerns around the use of embryonic cells. It is hoped that when stem cells are implanted into a patient, they will differentiate spontaneously into the desired mature tissue types and structures. It is also possible to differentiate stem cells into mature cell types in vitro. The success of stem cell therapy may depend on the degree of differentiation performed before implantation and the type of tissue being treated.

It is possible to create a range of 3-dimensional tissue constructs, including bioprinted tissues, tissue scaffolds, spheroids, organoids and assembloids, together termed SCTCs. SCTCs have varying complexity and may be freestanding, maintained on or within a biocompatible material. Current SCTCs range in size from <1 mm to several cm in diameter, allowing them to be injected into a patient or requiring a more invasive surgical application. SCTCs do not include dissociated cells, primary cells or complete organs.

One approach to creating structured tissues is organoids and assembloids. These 3-dimensional tissue structures self-organise and replicate the cell types and structures of more mature tissue. These tissues do not normally contain a supporting structure, but they can be grown into a scaffold or printed within bioinks [8, 9]. Protocols and culture kits are commercially available for creating a range of tissue types. Organoids and assembloids are being used to understand human development and disease, and for drug development and screening [10–12]. It has also been argued that they can be used to better define disease and for disease phenotypic screening [13].

There are many regenerative medicine applications for which SCTCs could be used. It may be possible to generate a mid-brain organoid containing dopaminergic cells and implant them into Parkinson’s disease patients. Patients with focal epilepsy may have the affected neural tissue resectioned; this may lead to behavioural deficits, such as loss of visual or verbal function if the lesion affected those areas. Currently, this tissue is not replaced, but it may be possible to regain functions by implanting a neural organoid. Patients suffering from age-related macular degeneration (AMD) and losing visual acuity have no available treatments; implanting retinal tissue may return visual function.

## Risks and benefits of dissociated stem cell and stem cell-derived product therapy

The collection and implantation of dissociated stem cells and their derived products can be performed by injection. This procedure poses minimal risk to the patient. When stem cells are placed into the patient, they can proliferate and differentiate into other tissue types. During human development, a range of cues direct the differentiation of cells into their correct type and arrangement. However, when stem cells are placed into a mature patient, the correct cues for their differentiation and integration may not be present. Thus stem cell differentiation may not occur, leading to formation of incorrect cell types, or tumours.

The benefits and risks of stem cell therapy are still being assessed. Clinical trials have been run for the implantation of embryonic and other stem cell or stem cell-derived products. To date, the FDA has only approved the use of blood-forming cells for treating cancer or immune disorders. Some other stem cell treatments currently available may be compliant with FDA rules but are not approved. Similar issues have been reported elsewhere, including in Europe and China [14, 15].

In July 2020, the FDA released a guidance document “Regulatory Considerations for Human Cells, Tissues, and Cellular and Tissue-Based Products (HCT/Ps): Minimal Manipulation and Homologous Use” to assist in classifying a tissue therapy [16]. HCT/Ps contain or consist of human cells or tissues intended for implantation, transplantation, infusion, or transfer into a human recipient, but not organs, blood or blood products, body secretions or animal tissue. Homologous use means the tissue performs the same basic function in the recipient as in the donor, but it may be in a different location in the body. The FDA and institutional review boards (IRBs) can provide guidance on whether a tissue has been minimally manipulated; however, this will not overcome offending stem cell clinics ignoring these rules.

According to this guidance, tissue collection, stem cell isolation and implantation could be classified as minimal manipulation, although the FDA has classified these ‘many steps’ as more than minimal manipulation, particularly cell purification and expansion, which involves culturing in media, and transportation between sites [17]. Under this regime, some stem cell therapies do not require a clinical trial, are not regulated or documented. They are classed as innovative ‘medical procedures’, rather than medical products requiring marketing approval. This designation also means they are not formally considered to be research, meaning they are not subject to oversight from research ethics committees or requirements to report results [18] unless clinicians are proactive in these regards. Subsequently, the rates and types of adverse events and patient benefits of these treatments are not known.

Without regulatory oversight, it is left to the clinician to determine if a tissue has been minimally manipulated. However, a clinician may have no understanding of tissue culture or recognize changes to the cell type. Clinics that offer direct-to-consumer stem cell treatments may also knowingly or unknowingly use cells in a non-homologous manner (e.g. adipose stem cells being injected into the eye). The added cost and administrative requirements in running a clinical trial and performing validation processes incentivises clinicians to claim their treatments are minimally manipulated, even when they may not be. Without regulatory oversight, it would be up to a patient to know what stem cells have been collected, if they have been manipulated and whether a clinician is using them in a homologous manner or not, which is clearly an impossible task.

Some severe adverse events following stem cell therapy at direct-to-consumer clinics have been recorded after patients presented to hospital for treatment [19–23].

While many treatments leading to severe adverse effects may meet the FDA’s minimal manipulation definition, they are not monitored to ensure they adhered to this standard. And without regulatory oversight or a clinical trial, their risk and benefit profile wasn’t determined.

Based on many trials [24–30], the known medical risks associated with dissociated stem cell and stem cell-derived product therapy are largely associated with cell proliferation, tumour or incorrect tissue formation, inflammation, surgically induced adverse events, and specific tissue reactions to the cell implantation including trauma. Other medical risks can arise from contaminants, or the materials and chemicals used in the preparation of the stem cells if they are not removed prior to implantation.

## Ethical issues of stem cell and stem cell-derived product therapy

Despite the limited benefit demonstrated by stem cell therapy, homologous, minimally manipulated stem cell therapies are permitted without a clinical trial by the FDA and other regulatory bodies (e.g. in Europe and Australia). The approach appears to reflect an expectation that such uses are low risk, but this is not borne out by existing data; or that patients should be able to decide for themselves, particularly where it concerns using their own cells [31]. But regulation of therapeutic goods exists in part because patients’ capacities to assess complex medical information can be limited. This can leave them open to exploitation, and decision-making can be distorted by hope, desperation or the way information is presented [32]. Despite moves by the FDA to clarify regulations, the current regulation removes the requirement for collecting risk and benefit data from minimally manipulated, homologous use stem cell therapy, placing participants at unnecessary health risk. Unproven treatments and clinical trials which charge participants a fee may enhance the risk of therapeutic misconception, exploit vulnerable patients, exclude less wealthy patients from participating, or support financially or medically unviable therapies. Stem cell and stem cell-derived therapies should have their risks and benefits assessed in clinical trials, as the majority of new treatments are assessed. The International Society of Stem Cell Research (ISSCR) and relevant agencies should consider banning stem cell therapies that are not assessed in a clinical trial. Only approved therapies should be offered to patients for a fee [33], in extension of the current ISSCR guidelines for Stem Cell Research and Clinical Translation [34].

Other ethical and regulatory issues relating to the collection, storage, research and medical use, and disposal of stem cells have been identified. The ISSCR Guidelines identify important issues and ways to address them [34]. In relation to clinical trials, however, the guidelines do not fully articulate that stem cell therapy may be irreversible, which may also prevent individuals from receiving any benefit from the trial or future therapies.

While the ISSCR Guidelines recommend recruiting a diverse range of trial participants they don’t take into account long-term risks and costs to the participant. For instance, a patient who develops tumours routinely after stem cell therapy may require repeated, lifelong surgical interventions for their removal. Or patients may become blind from stem cell therapy requiring lifelong assistance for navigation, changes to their living conditions, etc. Without ongoing support for trial participants at the conclusion of a clinical trial, less advantaged participants may suffer greater long-term costs and lifestyle difficulties. Greater on-going support should be offered to trial participants rather than excluding the less advantaged.

The clinical trial issues discussed in the ISSCR guidelines largely focus on currently available stem cell therapies. Issues identified with organoids and other stem cell-derived tissues are limited to their use in research and drug screening, not clinical use and regenerative medicine. Without guidelines over the use of SCTC therapy, patients may be placed at risk of serious harm. This raises the question, what new risks and ethical issues may arise from the use of SCTC therapy?

## Health risks of SCTC therapy

Compared to stem cell therapy, the likelihood of cell proliferation and tumour formation are reduced when implanting more mature tissue, while a number of new risks may arise. While the likelihood and severity of some risks may be low, the actual risk rate will not be known until further preclinical and clinical research has been undertaken, while other unknown risks of harm may not be recognized yet. It is critical that as many risks as possible are identified, so they can be investigated and minimised prior to first-in-human trials. It is also important to determine which risks can be investigated during preclinical trials and those that will remain poorly defined until first-in-human trials are complete. However, there is currently a very poor understanding of how relevant animal models are for determining risk of human stem cell and SCTC therapy.

Current SCTCs do not contain any vasculature [1]. As a result, some may develop a necrotic core during their formation. After implantation, if vasculature does not grow into the tissue, they may also fail to survive. The presence of necrotic tissue can affect surrounding tissue, requiring removal. This may exacerbate a patient’s trauma or disease, and if not treated can spread and lead to death. Organoids can also contain cysts. It is not known if these would persist after implantation or what impact they would have on tissue function. Possible risks from cysts could be sites for infection or patients experiencing pain. To reduce risks to patients, SCTCs should be assessed for necrosis and cysts, with affected tissue discarded before implantation. The development of vasculature into the implanted tissue and its survival should be monitored by MRI and CT scans so that preventative steps can be taken when necessary.

While SCTCs are structured, they are still not a true replication of normal tissue. While the complexity of tissue composition and structure will depend on the target organ, this raises questions around how personalised a tissue structure can be made and what is required to replicate normal tissue function? How specifically do tissue size, shape and structure need to be controlled? On the one hand, variation in structure of an islets of Langerhans SCTC implanted into the pancreas of a diabetic patient may have little impact on its function. In contrast, cortical organoids have a layering of cell types, but regions of the cortex are arranged in more specific patterns, such as columns in the visual cortex and long-range projections. If a patient has tissue resectioned to treat focal epilepsy, would a neural SCTC reorganise into the existing neural structure and create the necessary long-range projections? A tissue may also be implanted in the wrong orientation. Can the tissue function correctly with an atypical structure or incorrect placement? These issues must be assessed in animal models prior to commencing human trials; however, the relevance of animal models to humans is limited, and these issues will still remain during clinical trials.

Current SCTCs are not always “pure” and can contain off-target cell types. They also have a low reproducibility. Variations in tissue media or culturing protocol may have unexpected impacts on the tissue composition and structure. Clinicians performing stem cell implantation are not trained in tissue culture, and may not perform appropriate validation processes. There is a risk of wrong cell types being implanted (e.g. cortical neurons being present in a retinal SCTC) or the wrong differentiation protocol being used (e.g. a cortical rather than a mid-brain SCTC being created and implanted into the substantia nigra). The ratio of different cell types in a tissue may also be harmful, for instance, epilepsy is believed to be caused by an imbalance of excitatory and inhibitory cell types [35]. If an implanted neural SCTC is not balanced correctly, it may induce seizures. To reduce these risks, the composition of each tissue must be assessed by trained staff to validate their behaviour prior to implantation. Variations in tissue structure and composition should be documented and the impact correlated with patient outcomes. Staff trained in tissue culture must be included in the research and clinical phases of SCTC therapy.

The cells in SCTCs may not be fully mature [36]. The epigenetics of the tissue may also be altered during the culture protocol. It is unknown if the cells will mature correctly to the required cell types when implanted. It is also unknown if the cells will age in a natural manner and continue to function correctly. Cell development and integration may be affected by the age of the recipient, with better integration in young children compared to more mature adults and the elderly. There is a risk that over time the cells will die prematurely, or create misfolded proteins or plaques, leading to diseases, such as dementia or prion diseases. These degenerative diseases are poorly replicated in animal models, and animals don’t live as long as humans to assess ageing processes, so the determination of patient risk from preclinical trials will be very poor. And these adverse events can occur years after the conclusion of a clinical trial, so that they may not be detected. If misfolded proteins do occur, they may migrate to other tissues, and contaminate blood and tissue donations, subsequently affecting other people. Application of the precautionary principle may be to ban stem cell and SCTC recipients from making blood and tissue donations until these risks have been investigated. Patients receiving stem cell and SCTC therapies should be monitored for several years after implantation to assess tissue function, biofluid composition and rates of development of degenerative diseases.

The SCTC will have internal connectivity, but normal tissue has its own connectivity. It isn’t known if implanted tissue will connect to the rest of the body. An implanted tissue may be encapsulated by scar tissue. If the implanted tissue remains isolated, it may not provide any useful function. On the other hand, if it does connect to other tissues, it may disrupt already present connections and degrade surviving tissue functions. For instance, implanting dopaminergic cells into the midbrain may replace missing cells, but if they are not connected to the appropriate presynaptic cells, they will not release dopamine as required, which may exacerbate their symptoms or reduce the effectiveness of drug and other therapies.

Dissociated cells implanted in a patient may migrate large distances, while whole organ transplants are unlikely to migrate. In comparison, an implanted SCTC would be expected to migrate a small distance after implantation, but it is not clear how this would affect its connectivity and function. The impact of SCTC migration will depend on its overall structure and implanted location, both of which can be investigated in suitable animal models.

The implanted SCTC may interact with other medical interventions, leading to unexpected side effects. For instance, a Parkinson’s patient receiving a midbrain SCTC implant may be taking levodopa and have a deep brain stimulator implanted. Drugs and other therapies may impact on SCTC development, proliferation, integration and function. For instance, electrical stimulation of neural tissue has been performed in vitro and shown to affect cell function and structure [37].

The culture media used to create SCTCs varies from stem cell growth media, containing a range of chemicals and growth factors, and may contain foetal bovine serum or Matrigel (a highly variable media obtained from mouse sarcoma cells). Various materials are also used as scaffolds or bioinks. The safety of each culture media and support material must be determined and appropriate Good Manufacturing Practices (GMP) implemented for their use.

The 2013 FDA guidance “Preclinical Assessment of Investigational Cellular and Gene Therapy Products” and 2015 “Considerations for the Design of Early-Phase Clinical Trials of Cellular and Gene Therapy Products” provide some information on the development of new cellular or genetic therapies (CGT) [38, 39]. However, the only reference to the risks identified in these guidelines is to consider the fate of the cells post-administration (engraftment, migration, differentiation, tumorigenicity) in an animal model and no recommendations are given on managing or minimizing these risks. There may be a need to update current regulations and guidance documents to ensure SCTC risks of harm are appropriately assessed.

## Ethics issues of SCTCs: capacities, consciousness, moral status

Previous work addressing the implantation of human genes and tissues into animals to understand human development and disease raised concerns of humanization and the animals’ welfare needs [40–42]. It is possible that the implantation of SCTCs into a patient could create novel, emergent behaviour^1^ such as changes in cell or tissue function [43]. Emergent behaviours depend on the particular cells and their arrangement in a tissue; its properties are unpredictable and would only become evident during human trials. This may have an even greater possibility if allogenic tissue is implanted into a patient. For instance, if the tissue donor was susceptible to Alzheimer’s disease or epilepsy, the recipient may have an increased susceptibility to these diseases. The interaction of donor and recipient tissue may even enhance the susceptibility for these diseases or lead to novel disorders. This issue will likely be greater when larger amounts of donor tissue are implanted. If allogenic tissue is being used, it should be screened for disease. This raises issues where a disease is detected but the donor or their relatives are unaware of the condition and whether they should be informed [13]. There may also be requirements to monitor the donor’s health, to ensure they do not develop later-onset diseases. This would then require a non-anonymization and long-term follow-up of the donor with possible financial burdens for testing. Previously implanted patients would need to be informed of any changes in the donor’s health status and any adverse events in the patients noted. These may occur many years after the conclusion of a clinical trial, which may subsequently affect the risk/benefit profile of a therapy. Issues arising from the medical treatment following the conclusion of the trial and any insurance coverage can have significant financial and lifestyle implications to the patients, trial sponsor and others involved in the trial. Where possible, to minimise these issues, autologous stem cells should be used for regenerative medicine.

A significant amount of the ethics literature has focussed on neural organoids, but is relevant to other neural SCTCs. The creation of cortical organoids has raised speculative concerns about them developing some degrees of consciousness, self-awareness, advanced cognitive abilities or a capacity for suffering, capacities that are on many accounts linked to having (some) moral status [44]. There have been calls to identify methods and criteria for assessing sentience that can help set ethical rules for research conduct [45]. The National Academies of Sciences, Engineering and Medicine have developed guidelines specifically on the treatment and regulations of organoids and human/animal chimeras [4], but do not cover human implantation. This focus has no relevance to the majority of other non-neural SCTCs being generated and usually ignores other neural SCTCs. However should these hypothetical manifestations occur in future, they would affect the treatment of SCTCs, as such capacities may encourage people to regard them as subjects rather than objects. While current neural SCTCs lack sensory organs, they are unlikely to be conscious; however, forthcoming developments involving more complex tissues may introduce plausible related outcomes. Admittedly, the hypothesis of having an SCTC experiencing degrees of consciousness is ethically and philosophically loaded as there is no agreed definition of consciousness. Some words, such as “autonomous”, “thinking”, “feeling” and “sentience” may connote cognitive capabilities, as such, inducing emotional responses when protocols are being elaborated without much supportive evidence [46]. Ethically speaking, what does it mean conceptually for a bundle of in vitro *je ne sais quoi* cells to be potentially conscious and sentient? Differentiating SCTC potential by known functions and applications, rather than speculative cognitive capacities would avoid an unjustified moratorium on their development. There is also disagreement about which capacities, if any, indicate moral status, with some arguing that consciousness on its own has no moral significance, rather the psychological capacities that enable high-level cognitive sophistication are more important [47]; though it is a minority view, some ground moral status in features other than capacities entirely [48]. Furthermore, there is currently no way of measuring if a SCTC is conscious, self-aware, has advanced cognitive abilities or a capacity for suffering [49]. Subsequently, a person growing SCTCs would not know if it has these capacities.

Notwithstanding these qualifications, if a SCTC does possess some of these capacities, and is implanted into a patient, how would it impact on the tissue and the patient? The tissue may integrate into the patient and lose any capacities. If the tissue is thought to be a conscious or self-aware individual entity, this integration might be akin to causing the death of an entity with moral status, making it unethical to implant it into a patient.

This claim would give tissues with relevant capacities something approaching the moral status sometimes accorded to an embryo. But while an embryo could develop into an adult, disembodied tissue is not able to develop into a mature individual. From this viewpoint, the capacity for autonomous existence becomes a further criterion for full moral status. Since the tissues could not meet this criterion, their loss of individual status would be less ethically concerning. It has been argued that an entity that has no autonomous existence should have no more protection than non-human animals used in research [50].

Alternatively, implanting a tissue with capacities may result in a merging with the patients’ capacity. In this instance, there is a risk of psychological harms and changes to the patients’ personality as the tissue integrates into their brain. The assessment of psychological risk is not normally undertaken in a clinical trial, we recommend the inclusion of a psychologist to determine any potential adverse neuropsychiatric outcomes such as personality changes, depression, etc. As with any object or tissue inserted into the body, in particular the brain, many plausible scenarios may occur as a result of the implantation [51]. These issues may occur regardless of the capacity of the implanted tissue. On the one hand, the migration, integration and immune response of tissue in some specific brain regions may alter cognitive faculties and induce unwanted neuropsychiatric effects including self-estrangement - as observed with other invasive brain interventions [52]. On the other hand, their removal could exacerbate existing psychiatric problems [52]. When looking at neural device implantation, such as deep brain stimulation, there is a significant body of evidence of patients/families reporting postoperative maladaptations, including reports of personality changes, depression and suicidal tendencies [53]. If a patient experiences a general increase in their sexual arousal and activity, tangential with augmented impulsivity following implantation, it may call into question whether this patient is ultimately responsible for some of their behaviours and actions [54], especially if the implantation is correlated with a disruption of psychological continuity influencing competence, accountability and consent [55].

To minimise the issues of tissue capacity, smaller neural SCTCs that are less likely to display consciousness should be used for regenerative medicine. Further efforts are needed to understand how tissue and individuals develop these capacities. As noted above, the issue of consciousness and moral status only applies to neural tissue. These issues will be avoided by implanting other tissues, and subsequently they should be the initial target when assessing the risks and benefits of SCTCs for regenerative medicine.

Reproductive tissue could also be created from stem cells. The formation of gamete forming tissue may assist with patients suffering from reproductive diseases or trauma. However, the function of the tissue is not known, and would have significant risks that may affect offspring. Subsequently, the use of stem cells to create gamete-forming tissue should be banned for now, or until it has been proven safe and effective. However, the testing of reproductive tissue would be further hindered as the ISSCR and NAS guidelines recommend limits on breeding and foetus development with human/animal chimeras.

## Ethical issues of SCTCs: legal status and ownership

In regards to the regulatory issues of stem cell therapies discussed above, SCTCs are unlikely to involve minimal manipulation. It is possible some clinics might try to exploit regulatory gaps, but the possibility for this is lower than for other stem cell treatments. This is a positive, but leads to other questions about the commercial status and ownership of the tissues. Donated blood, tissues and organs cannot proliferate and have a limited lifetime outside a body; in contrast stem cells and SCTCs can proliferate and differentiate into a large number of tissues and be maintained indefinitely in a lab. Insofar as the tissues are more than minimally manipulated, and so subject to research and marketing regulations, they are also constructed by regulatory systems as marketable commodities. It is the commercial status of a product that triggers marketing regulation, and subsequent research regulation. Yet most jurisdictions prohibit treating body parts — if such these are — as commodities. These prohibitions have been developed to avoid a situation where people sell their organs for profit and associated possibilities for exploitation of the socioeconomically disadvantaged. However, in the case of stem cell donations (as with blood donations) these would require very small tissue biopsies with minimal health impact to the donor. Regardless, some argue that selling body parts would undermine human dignity [56]. These issues have led to the conceptualisation of donated tissue as a gift, rather than a commodity [57], tissue donation organisations may only charge a fee to cover the costs of their service. Cells obtained from some donors who have provided specific consent may be sold for profit by biotechnology companies, solely for research purposes, these cells are not to be used for commercial use.

Legal precedents indicate that people do not by default own their tissues once they have been removed from the body, especially where the tissues have been manipulated by researchers in many jurisdictions, including Australia, France, the UK and USA [58–62].

This suggests that due to modifications made to primary cells and the unknown potential benefit of the tissues at the time of their donation, tissues created from patient-derived stem cells would not be considered their property and might be considered the property of researchers, clinics, or institutions. But SCTCs might be considered more like ‘body parts’ than other, renewable kinds of biological tissues that can be donated (including for payment in the USA) such as blood and gametes. This militates against treating them as commercial objects and might impact on public acceptance of the technologies. If researchers use cells more broadly than for the patients’ own treatments in research studies, or treatments are translated into clinical practice, there may be a need to clarify legislation surrounding the sale of body parts, particularly if allogeneic cells are used. It is also possible (including in autologous uses) that clinics could present uses of SCTCs as procedures rather than ‘medical products’, which will also require regulatory clarification.

Currently, tissue donors must provide consent to their tissue being used for various research or medical purposes. The ISSCR Guidelines provide a list of informed consent considerations which can include the use of stem cells in research or clinical therapy, their genetic modification, tissue and data storage, who can use the cells, donor confidentiality, rights to know what the cells are used for, commercial use of the cells or their research outcomes, prevention for use in reproductive purposes and that donation or non-donation doesn’t affect clinical care [34]. Since many patients reportedly assume that they own their cells by default [17], disclosure around potential commercial use should be particularly explicit. This should also form part of the ISSCR’s standard for informed consent in clinical care [63].

The potential for tissue to develop capacities is not covered at all in the ISSCR guidelines. Donors should be given the right to consent for their tissue being used to develop with capacities. They may then retain or assign rights over the usage of a tissue with these capacities. While highly speculative, at what point does the tissue become an individual entity with its own rights [64]? As the tissue does not have the capacity to provide consent for its usage in research or therapy, it may be better to view the donor, researcher or clinician as a guardian of the tissue, in which case they have the legal authority to decide on the tissue’s fate, irrespective of its capacity.

The current knowledge of the risks and benefits of stem cell therapy and the relevance of animal studies raises questions around how clinical trials for SCTC therapy should be structured and what level of preclinical research is required.

## Clinical trial and regulatory paradigm for testing and market approval of SCTCs

The FDA offer different clinical trial structures for assessing non-traditional medical therapies [65]. The most appropriate trial structure and preclinical testing requirements for a new therapy can be discussed with the FDA. However, the guidelines and regulations often lag behind current scientific knowledge. While clinical trials for SCTCs are not unique, being similar to stem cell trials and organ transplants, these trial structures may still not be optimal. Clearer guidance on trial structure and preclinical testing requirements would assist researchers and clinicians in developing new therapies. It would also ensure the risks to trial participants are minimised and the trial benefits are maximised. The following recommendations are applicable to clinical trials of both stem cell and SCTC therapies.

The relevance of animal studies for stem cell and SCTC therapy is largely unknown. Some animals have greater abilities to regenerate tissue and have different immune systems, vasculature and nerve growth, physiology and metabolism to humans. Subsequently, the survival, integration and function of animal or human stem cells and SCTCs implanted into an animal model may be very different from in a human.

Traditional preclinical studies on animals and cell lines assess the efficacy, toxicity, dose response, pharmacokinetics, pharmacodynamics and mode of action of a therapy, and are used to guide which parameters to monitor, who are the most eligible patients, and what is the best treatment delivery method for first-in-human studies. The first-in-human studies are then used to validate any preclinical studies and better define the phase 1 trial structure. However, the high level of uncertainty of preclinical data relevance of risk and benefit to first-in-human trial participants places them at very high risk of harm. Yet positive data from preclinical studies may give clinicians and review boards a false understanding of treatment risk and benefit. This may lead to overly enthusiastic portrayal of the treatment to potential trial participants, generating a false hope of therapeutic benefit and low treatment risk [66]. It may also needlessly harm large numbers of animals testing potential therapies which have low relevance to humans.

What details can be obtained from a preclinical study of stem cell and SCTC therapy? Current FDA guidelines for preclinical assessment of gene and cellular therapies include investigating the animals immune response, the animal and implanted tissue survival, stem cell proliferation, differentiation, engraftment and migration, and determining a suitable initial cell dosage level [38, 39]. The animal testing should achieve an understanding of the biological plausibility of the therapy with characterisation including morphology, functional and animal behavioural changes. Safety should be assessed by toxicology studies. And if a scaffold is implanted, it should be adequately characterized for composition, degradation profile, biomechanical performance, and biocompatibility.

Where appropriate, a preclinical study for stem cell and SCTC therapy should define the cell source, differentiation method, tissue plating, culture timing and media composition; it should demonstrate consistent tissue yield, purity and phenotype. Animal studies should assess the impact of incorrect tissue placement, orientation and composition on tissue and animal survival and function. The effect of necrotic tissue and cysts should be determined and the impact on tissue and animal survival when vasculature fails to grow into the implanted tissue. Changes in tissue maturity, structure, function, survival and integration should be assessed over long-term implantation. Different aged animals may be implanted to determine the impact on tissue development. And biofluid samples may be assessed for changes after implantation. Implanted animals should also be given other disease-specific standard treatments such as immunosuppressants, drugs or electrical stimulation to determine the impact on implanted tissue function and survival, and changes to the other treatments performance.

These preclinical studies will provide some guidance on how a stem cell or SCTC will perform in a first-in-human study. However, due to differences in genetics and target tissue, the function may vary in every person. These studies also have limited benefit in understanding how autologous tissue will function. The poor relevance of the animal studies to humans precludes the need to test every autologous tissue before clinical use. Preclinical studies would only be required before a first-in-human clinical trial for a particular tissue protocol.

Once a stem cell or SCTC therapy has proceeded to a clinical trial, how should the trial be structured? Trials will likely need to incorporate efficacy as well as safety endpoints in ways that are fair and respectful to research trial participants [67]. The question is how to manage uncertainties related to risk of harm, in particular with custom made tissues. Does a precautionary approach to the risk-benefit ratio work? Autologous SCTCs may be fabricated on a tailor-made ‘per-patient’ basis, and so a precautionary approach might be less relevant. As such, it would be of limited clinical value and unethical to first test safety in a randomised clinical trial on a different population of non-specific subjects. This may entail that fabricating unique personalised SCTC treatments on demand from one’s own stem cells will unlikely require a prior randomized safety and efficacy trial on other patients as the relevance is limited, aside from standardised criteria and protocols for building the tissues. Accordingly, a patient waiting for a SCTC would likely serve as their own test subject or a ‘guinea pig’ for their therapy [67]. Where possible, there should be a standardisation of primary and secondary endpoints for different regenerative medicine targets to enable comparison of safety and efficacy.

Patient recruitment should be staggered with an individual patient implanted first, the risks and benefits of the stem cell or SCTC therapy monitored over a sufficiently long time period [68]. If the treatment was successful, a second patient can be enrolled. If the treatment was not successful, then the protocol may be modified to address any known issues before enrolling a second patient. Given the unknowns surrounding both benefits and iatrogenic harms, patient selection should favour those with no other treatment options. At the conclusion of the trial, all data should be published so that no other patients are needlessly harmed in similar future trials and the knowledge obtained from the patients provides benefit to society. Trial participants should be made aware of sponsors that have not published previous trial data, as this may suggest their involvement will lead to no benefit to society. Our previous work on restructuring clinical trials based on treatment risk provides further information and examples to aid IRBs in evaluating the risk and efficacy of a novel therapy [68].

The risk of neural and reproductive regenerative medicine therapies is greater than other tissues, which may suggest they should not be assessed initially. However, trial sponsors are not organised to assess new medical therapies beginning with the lowest risk. Trial sponsors will likely target diseases with greater prevalence in first world countries. The cost of SCTC therapy is very high, so it may not receive reimbursement. This raises concerns around equity of trial and treatment access. Where possible, stem cell and SCTC therapy should be provided through the public health care system to ensure equal access regardless of patient or disease.

The choice of population group for enrolling in a clinical trial is also complicated. Due to differences in disease presentation, treatment risk and methodology, it is not possible to provide a blanket rule for patient selection and recruitment of every stem cell and SCTC trial. Patients requiring tissue replacement may suffer degenerative disease or severe trauma with no treatment, including significant mental trauma, and may be desperate for a therapeutic option. And there is the possibility of testing underage patients or those with mental disabilities that affect their ability to provide informed consent. Certain diseases are less likely to include people that are underage or have mental disabilities and should be the initial target for testing stem cell and SCTC therapies. Further discussion over the choice of patients for first-in-human clinical trials of high-risk therapies is detailed in reference [68].

While there is guidance to protect patients enrolling in a clinical trial, the principle investigator takes responsibility for conducting ethical research and is accountable for noncompliance and misconduct. The specific responsibilities for principle investigators, sponsors, IRBs, trial participants and regulatory agencies are numerous and further information can be obtained from other sources [69–71].

## Conclusions

Stem cell-derived organoid and tissue therapy has the potential to treat many disorders that have no available treatments. There are numerous health and ethical risks associated with SCTC therapy which differ from stem cell or stem cell-derived product therapy. These risks are not addressed in current guidelines or regulations. There are numerous unknowns in using SCTCs for regenerative medicine including possibilities of harms from tissue necrosis or cysts; differences between cultured and biological tissue structure or function; unknown capacities to connect to and interact with surrounding tissues, and their variability, low reproducibility and immaturity. Preclinical animal studies of SCTC implants are poor models of human regenerative therapy. Subsequently, patients may be placed at significant risk of harm. In addition, although this form of regenerative medicine may avoid ethical issues related to organ donation or the use of embryonic stem cells, it generates novel issues relating to the moral status of the SCTCs, whether they are considered subjects with some intrinsic moral status, or as something similar to a body part. Patients receiving SCTC therapy should be monitored for long periods to assess risks and benefits. The use of allogenic tissue raises extra risks, so where possible, autologous tissue should be used. Clinical trials should be run in a staggered process to ensure patients are not placed in needless harm.

## Data Availability

Not applicable.

## Footnotes

1 Emergent behaviours arise through the interaction of units in a complex system and are not attributed to an individual unit. For example novel properties arising from the interaction of cells in tissue, and not those attributed to an individual cell, such as an epileptic seizure.
